# 2,2′-[(3a*RS*,7a*RS*)-Perhydro­benz­imid­azole-1,3-di­yl)bis­(methyl­ene)]diphenol

**DOI:** 10.1107/S1600536810009918

**Published:** 2010-03-27

**Authors:** Augusto Rivera, Diego Quiroga, Jaime Ríos-Motta, Michal Dušek, Karla Fejfarová

**Affiliations:** a Departamento de Química, Universidad Nacional de Colombia, Bogotá, AA 14490, Colombia; b Institute of Physics, Na Slovance 2, 182 21 Praha 8, Czech Republic

## Abstract

The molecular structure of the title compound, C_21_H_26_N_2_O_2_, shows two intra­molecular O—H⋯N hydrogen-bonding inter­actions. In the crystal structure, mol­ecular chains are formed along the *c* axis through weak C—H⋯O inter­actions. Neighbouring chains are weakly associated along the *a* axis *via* C—H⋯π inter­actions.

## Related literature

For a related structure, see: Rivera *et al.* (2009 ▶). For uses of di-Mannich bases, see Mitra *et al.* (2006 ▶); Elias *et al.* (1997 ▶).
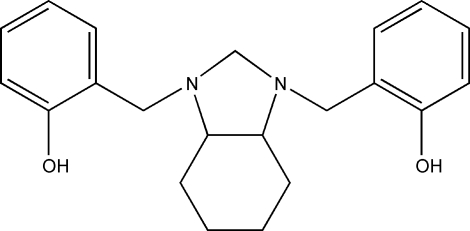


## Experimental

### 

#### Crystal data

C_21_H_26_N_2_O_2_
*M* *_r_* = 338.5
Triclinic, 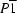

*a* = 5.5177 (1) Å
*b* = 12.0432 (4) Å
*c* = 14.3752 (4) Å
α = 69.705 (3)°
β = 89.341 (2)°
γ = 81.751 (2)°
*V* = 885.92 (5) Å^3^
*Z* = 2
Cu *K*α radiation
μ = 0.65 mm^−1^
*T* = 120 K
0.24 × 0.21 × 0.19 mm

#### Data collection

Oxford Diffraction Xcalibur, diffractometer with an Atlas (Gemini ultra Cu) detector
Absorption correction: multi-scan (*CrysAlis PRO*; Oxford Diffraction, 2009 ▶) *T*_min_ = 0.774, *T*_max_ = 1.000
11989 measured reflections
3023 independent reflections
2685 reflections with *I* > 3σ(*I*)
*R*_int_ = 0.018

#### Refinement

*R*[*F* ^2^ > 2σ(*F* ^2^)] = 0.038
*wR*(*F* ^2^) = 0.118
*S* = 2.41
3023 reflections
232 parameters
H atoms treated by a mixture of independent and constrained refinement
Δρ_max_ = 0.25 e Å^−3^
Δρ_min_ = −0.19 e Å^−3^

### 

Data collection: *CrysAlis CCD* (Oxford Diffraction, 2009 ▶); cell refinement: *CrysAlis RED* (Oxford Diffraction, 2009 ▶); data reduction: *CrysAlis RED*; program(s) used to solve structure: *SIR2002* (Burla *et al.*, 2003 ▶); program(s) used to refine structure: *JANA2006* (Petříček *et al.*, 2006 ▶); molecular graphics: *DIAMOND* (Brandenburg & Putz, 2005 ▶); software used to prepare material for publication: *JANA2006*.

## Supplementary Material

Crystal structure: contains datablocks global, I. DOI: 10.1107/S1600536810009918/tk2643sup1.cif

Structure factors: contains datablocks I. DOI: 10.1107/S1600536810009918/tk2643Isup2.hkl

Additional supplementary materials:  crystallographic information; 3D view; checkCIF report

## Figures and Tables

**Table 1:** Hydrogen-bond geometry (Å, °) *Cg*1 and *Cg*2 are the centroids of the C3–C8 and C16–C21 rings, respectively.

*D*—H⋯*A*	*D*—H	H⋯*A*	*D*⋯*A*	*D*—H⋯*A*
O1—H1O⋯N1	0.85 (2)	1.97 (2)	2.7096 (14)	146 (2)
O2—H2O⋯N2	0.86 (2)	1.91 (2)	2.6894 (14)	150 (2)
C10—H10*a*⋯O1^i^	0.96	2.64	3.5666 (17)	163
C13—H13*a*⋯O2^ii^	0.96	2.63	3.5458 (17)	160
C10—H10*b*⋯*Cg*1^iii^	0.96	2.94	3.5885 (13)	126
C12—H12*b*⋯*Cg*2^iv^	0.96	2.93	3.7022 (16)	139

Symmetry codes: (i) 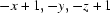; (ii) 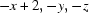; (iii) 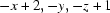; (iv) 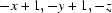.

**Fig. 1.**
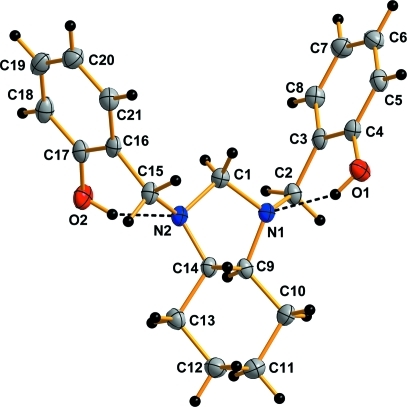
The molecular structure of (I), showing the atomic numbering scheme with atomic displacement ellipsoids drawn at the 50% probability level. The dashed lines indicates intramolecular O–H···N hydrogen bonds.

**Fig. 2.**
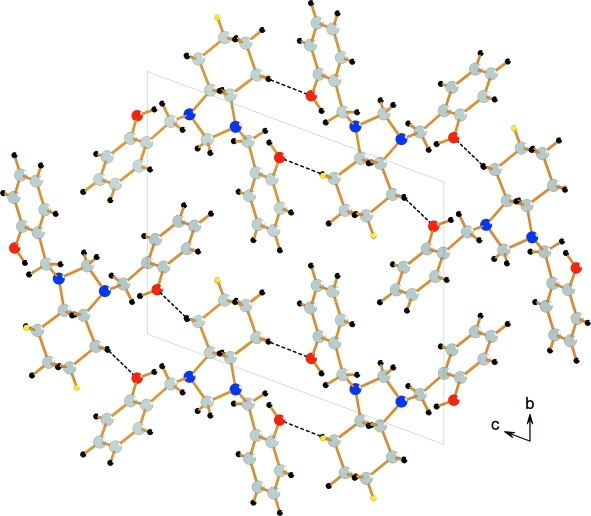
Packing diagram for (I) with intermolecular interactions drawn as dashed lines.

## supplementary crystallographic information

### Comment

Mannich bases are versatile synthetic intermediates and are used as model systems for the study of intramolecular hydrogen bonding and proton transfer (Mitra *et al.* 2006; Elias *et al.* 1997). The presence of these interactions is undoubtedly one of the essential factors contributing to their highly thermodynamic stability. Recently, we used (2*R*,7R,11*S*,16S)-1,8,10,17-tetraazapentacyclo[8.8.1.1^8,17^. 0^2,7^.0^11,16^]icosane as precursor for a di-Mannich base (Rivera *et al.* 2009). The X-ray analysis showed the N lone pairs to be anti-axial and both N atoms to be sufficiently basic to form intramolecular hydrogen bonds. In continuation of our research program on the structure, properties, and reactivity of aminal cages (pre-formed Mannich reagents), we report here the synthesis and crystal structure of the title compound, (I).

Compound (I) features intramolecular hydrogen bonds O—H···N, Fig. 1. The bond lengths are normal and comparable to the corresponding values observed in the related structure of 2,2'-(3aR,7aR/3aS,7aS)-hexahydro-1*H*-benzo[*d*]imidazole-1,3(2*H*)-diyl)-bis(methylene)-bis(4-methyl-phenol) (Rivera *et al.* 2009).

The crystal packing (Fig 2) displays weak intermolecular C–H···O interactions (Table 1) that link pairs of enantiomers alternately to form a racemic chain along the *c* axis. Chains are linked along the *a* direction by C–H···π interactions (Table 1).

### Experimental

A solution of (2*R*,7R,11*S*,16S)-1,8,10,17-tetraazapentacyclo[8.8.1.1^8,17^. 0^2,7^.0^11,16^]icosane (276 mg, 1.00 mmol) in dioxane (3 ml) and water (4 ml), prepared beforehand following previously described procedures, was added dropwise into a dioxane solution (3 ml) containing two equivalents of phenol (188 mg, 2.00 mmol) in a two-necked round-bottomed flask. The mixture was refluxed for about 12 h. The solvent was evaporated under reduced pressure until a sticky residue appeared. The product was purified by chromatography on a silica column, and subjected to gradient elution with benzene:ethyl acetate (yield 21%, M.pt. = 413–414 K). Single crystals of racemic (I) were grown from a CHCl_3_ solution by slow evaporation of the solvent at room temperature over a period of about 2 weeks.

### Refinement

The C-bound H atoms were geometrically placed (C-H = 0.96 Å) and refined as riding with *U*_iso_(H) = 1.2*U*_eq_(C). The positions of the hydroxyl-H atoms were refined with *U*_iso_(H) = 1.2*U*_eq_(O).

### Crystal data

**Table:**

C_21_H_26_N_2_O_2_	*Z* = 2
*M**_r_* = 338.5	*F*(000) = 364
Triclinic, *P*1	*D*_x_ = 1.268 Mg m^−^^3^
Hall symbol: -P 1	Cu *K*α radiation, λ = 1.54184 Å
*a* = 5.5177 (1) Å	Cell parameters from 9142 reflections
*b* = 12.0432 (4) Å	θ = 3.3–65.4°
*c* = 14.3752 (4) Å	µ = 0.65 mm^−^^1^
α = 69.705 (3)°	*T* = 120 K
β = 89.341 (2)°	Prism, colorless
γ = 81.751 (2)°	0.24 × 0.21 × 0.19 mm
*V* = 885.92 (5) Å^3^	

### Data collection

**Table:**

Oxford Diffraction Xcalibur, diffractometer with an Atlas (Gemini ultra Cu) detector	3023 independent reflections
Radiation source: X-ray tube	2685 reflections with *I* > 3σ(*I*)
mirror	*R*_int_ = 0.018
Detector resolution: 10.3784 pixels mm^-1^	θ_max_ = 65.8°, θ_min_ = 3.3°
Rotation method data acquisition using ω scans	*h* = −6→6
Absorption correction: multi-scan (*CrysAlis PRO*; Oxford Diffraction, 2009)	*k* = −13→13
*T*_min_ = 0.774, *T*_max_ = 1.000	*l* = −16→16
11989 measured reflections	

### Refinement

**Table:**

Refinement on *F*^2^	98 constraints
*R*[*F*^2^ > 2σ(*F*^2^)] = 0.038	H atoms treated by a mixture of independent and constrained refinement
*wR*(*F*^2^) = 0.118	Weighting scheme based on measured s.u.'s *w* = 1/[σ^2^(*I*) + 0.0016*I*^2^]
*S* = 2.41	(Δ/σ)_max_ = 0.006
3023 reflections	Δρ_max_ = 0.25 e Å^−^^3^
232 parameters	Δρ_min_ = −0.19 e Å^−^^3^
0 restraints	

### Special details

**Table:**

Refinement. The refinement was carried out against all reflections. The conventional *R*-factor is always based on *F*. The goodness of fit as well as the weighted *R*-factor are based on *F* and *F*^2^ for refinement carried out on *F* and *F*^2^, respectively. The threshold expression is used only for calculating *R*-factors etc. and it is not relevant to the choice of reflections for refinement.The program used for refinement, Jana2006, uses the weighting scheme based on the experimental expectations, see _refine_ls_weighting_details, that does not force *S* to be one. Therefore the values of *S* are usually larger than the ones from the SHELX program.

### Fractional atomic coordinates and isotropic or equivalent isotropic displacement parameters (Å^2^)

**Table:**

	*x*	*y*	*z*	*U*_iso_*/*U*_eq_	
O1	0.55804 (17)	0.14020 (9)	0.44644 (7)	0.0291 (4)	
O2	0.83868 (17)	0.18264 (9)	−0.03369 (7)	0.0305 (4)	
N1	0.79361 (18)	0.08554 (9)	0.29799 (7)	0.0202 (4)	
N2	0.62975 (19)	0.10318 (10)	0.14258 (7)	0.0213 (4)	
C1	0.6914 (2)	0.17421 (12)	0.20218 (9)	0.0226 (5)	
C2	0.9951 (2)	0.12344 (12)	0.33988 (9)	0.0226 (5)	
C3	0.9035 (2)	0.23158 (11)	0.36752 (9)	0.0216 (4)	
C4	0.6882 (2)	0.23380 (12)	0.42039 (9)	0.0227 (4)	
C5	0.6072 (2)	0.33136 (12)	0.44923 (9)	0.0261 (5)	
C6	0.7390 (2)	0.42599 (13)	0.42654 (10)	0.0289 (5)	
C7	0.9518 (2)	0.42527 (13)	0.37409 (10)	0.0289 (5)	
C8	1.0308 (2)	0.32828 (12)	0.34497 (10)	0.0254 (5)	
C9	0.8513 (2)	−0.02791 (11)	0.27990 (9)	0.0210 (4)	
C10	0.8803 (2)	−0.14204 (12)	0.36961 (9)	0.0265 (5)	
C11	0.9041 (3)	−0.24770 (13)	0.33239 (10)	0.0295 (5)	
C12	0.6923 (3)	−0.23872 (12)	0.26116 (10)	0.0295 (5)	
C13	0.6592 (2)	−0.11997 (12)	0.17294 (10)	0.0259 (5)	
C14	0.6331 (2)	−0.01887 (11)	0.21374 (9)	0.0207 (4)	
C15	0.4060 (2)	0.15656 (12)	0.07972 (9)	0.0231 (5)	
C16	0.4414 (2)	0.27197 (12)	−0.00170 (9)	0.0220 (4)	
C17	0.6553 (2)	0.27821 (12)	−0.05575 (9)	0.0242 (5)	
C18	0.6825 (3)	0.38203 (13)	−0.13478 (10)	0.0297 (5)	
C19	0.4978 (3)	0.47854 (13)	−0.16005 (10)	0.0322 (5)	
C20	0.2872 (3)	0.47453 (13)	−0.10655 (10)	0.0321 (5)	
C21	0.2613 (2)	0.37168 (13)	−0.02762 (10)	0.0273 (5)	
H1a	0.544692	0.22078	0.213055	0.0271*	
H1b	0.814039	0.221818	0.169948	0.0271*	
H2a	1.119524	0.142433	0.292045	0.0271*	
H2b	1.066463	0.059026	0.397776	0.0271*	
H5	0.459427	0.332936	0.484953	0.0313*	
H6	0.683126	0.492726	0.447191	0.0347*	
H7	1.042987	0.491174	0.358217	0.0346*	
H8	1.177255	0.328004	0.308288	0.0304*	
H9	1.010365	−0.035487	0.253016	0.0252*	
H10a	0.737945	−0.142807	0.408735	0.0318*	
H10b	1.025945	−0.147212	0.407689	0.0318*	
H11a	0.912941	−0.321113	0.388014	0.0353*	
H11b	1.056388	−0.252589	0.300031	0.0353*	
H12a	0.543303	−0.246619	0.296477	0.0354*	
H12b	0.720963	−0.304289	0.237045	0.0354*	
H13a	0.800881	−0.116292	0.133191	0.031*	
H13b	0.513586	−0.113908	0.134504	0.031*	
H14	0.478413	−0.028884	0.244425	0.0249*	
H15a	0.273969	0.172023	0.119698	0.0277*	
H15b	0.361755	0.10108	0.050763	0.0277*	
H18	0.829452	0.386296	−0.171479	0.0356*	
H19	0.515368	0.549372	−0.215424	0.0386*	
H20	0.160216	0.542534	−0.124022	0.0386*	
H21	0.115837	0.369273	0.009909	0.0328*	
H1o	0.604 (3)	0.0973 (15)	0.4118 (12)	0.0349*	
H2o	0.810 (3)	0.1344 (15)	0.0240 (13)	0.0366*	

### Atomic displacement parameters (Å^2^)

**Table:**

	*U*^11^	*U*^22^	*U*^33^	*U*^12^	*U*^13^	*U*^23^
O1	0.0304 (5)	0.0306 (6)	0.0309 (6)	−0.0103 (4)	0.0112 (4)	−0.0146 (4)
O2	0.0248 (5)	0.0374 (6)	0.0250 (5)	−0.0010 (4)	0.0063 (4)	−0.0072 (4)
N1	0.0204 (5)	0.0221 (6)	0.0178 (5)	−0.0029 (4)	−0.0004 (4)	−0.0065 (4)
N2	0.0215 (6)	0.0241 (6)	0.0173 (5)	−0.0028 (4)	−0.0008 (4)	−0.0061 (4)
C1	0.0236 (7)	0.0238 (7)	0.0200 (7)	−0.0034 (5)	0.0002 (5)	−0.0069 (5)
C2	0.0198 (6)	0.0279 (7)	0.0216 (7)	−0.0042 (5)	0.0002 (5)	−0.0101 (5)
C3	0.0204 (6)	0.0270 (7)	0.0161 (6)	−0.0012 (5)	−0.0029 (5)	−0.0067 (5)
C4	0.0230 (6)	0.0269 (7)	0.0177 (6)	−0.0045 (5)	−0.0006 (5)	−0.0068 (5)
C5	0.0239 (7)	0.0317 (8)	0.0231 (7)	−0.0014 (5)	0.0020 (5)	−0.0112 (6)
C6	0.0321 (7)	0.0271 (8)	0.0282 (7)	0.0006 (6)	−0.0014 (6)	−0.0124 (6)
C7	0.0305 (7)	0.0278 (7)	0.0294 (7)	−0.0085 (6)	−0.0003 (6)	−0.0098 (6)
C8	0.0215 (7)	0.0304 (8)	0.0239 (7)	−0.0042 (5)	0.0012 (5)	−0.0089 (6)
C9	0.0195 (6)	0.0251 (7)	0.0198 (6)	−0.0033 (5)	0.0027 (5)	−0.0097 (5)
C10	0.0292 (7)	0.0266 (7)	0.0214 (7)	−0.0021 (6)	−0.0025 (5)	−0.0061 (6)
C11	0.0333 (8)	0.0239 (7)	0.0285 (7)	−0.0024 (6)	−0.0004 (6)	−0.0066 (6)
C12	0.0320 (8)	0.0258 (8)	0.0321 (7)	−0.0041 (6)	0.0000 (6)	−0.0118 (6)
C13	0.0254 (7)	0.0292 (7)	0.0245 (7)	−0.0025 (5)	−0.0020 (5)	−0.0119 (6)
C14	0.0193 (6)	0.0239 (7)	0.0188 (6)	−0.0034 (5)	0.0033 (5)	−0.0071 (5)
C15	0.0197 (6)	0.0295 (7)	0.0186 (6)	−0.0031 (5)	0.0003 (5)	−0.0068 (5)
C16	0.0225 (6)	0.0276 (7)	0.0160 (6)	−0.0050 (5)	−0.0022 (5)	−0.0073 (5)
C17	0.0226 (7)	0.0315 (8)	0.0197 (6)	−0.0043 (5)	−0.0012 (5)	−0.0103 (6)
C18	0.0279 (7)	0.0399 (8)	0.0220 (7)	−0.0132 (6)	0.0034 (5)	−0.0086 (6)
C19	0.0403 (8)	0.0296 (8)	0.0245 (7)	−0.0136 (6)	−0.0028 (6)	−0.0034 (6)
C20	0.0354 (8)	0.0282 (8)	0.0297 (8)	−0.0025 (6)	−0.0045 (6)	−0.0069 (6)
C21	0.0245 (7)	0.0331 (8)	0.0241 (7)	−0.0029 (6)	0.0008 (5)	−0.0104 (6)

### Geometric parameters (Å, °)

**Table:**

O1—C4	1.3640 (17)	C9—H9	0.96
O1—H1o	0.85 (2)	C10—C11	1.531 (2)
O2—C17	1.3672 (15)	C10—H10a	0.96
O2—H2o	0.860 (16)	C10—H10b	0.96
N1—C1	1.4766 (14)	C11—C12	1.530 (2)
N1—C2	1.4691 (19)	C11—H11a	0.96
N1—C9	1.4682 (19)	C11—H11b	0.96
N2—C1	1.476 (2)	C12—C13	1.5360 (17)
N2—C14	1.4686 (15)	C12—H12a	0.96
N2—C15	1.4657 (15)	C12—H12b	0.96
C1—H1a	0.96	C13—C14	1.513 (2)
C1—H1b	0.96	C13—H13a	0.96
C2—C3	1.508 (2)	C13—H13b	0.96
C2—H2a	0.96	C14—H14	0.96
C2—H2b	0.96	C15—C16	1.5112 (17)
C3—C4	1.4045 (18)	C15—H15a	0.96
C3—C8	1.387 (2)	C15—H15b	0.96
C4—C5	1.391 (2)	C16—C17	1.4021 (18)
C5—C6	1.382 (2)	C16—C21	1.3873 (18)
C5—H5	0.96	C17—C18	1.3932 (17)
C6—C7	1.388 (2)	C18—C19	1.3774 (19)
C6—H6	0.96	C18—H18	0.96
C7—C8	1.385 (2)	C19—C20	1.383 (2)
C7—H7	0.96	C19—H19	0.96
C8—H8	0.96	C20—C21	1.3836 (18)
C9—C10	1.5132 (16)	C20—H20	0.96
C9—C14	1.5110 (18)	C21—H21	0.96
			
C4—O1—H1o	108.2 (12)	H10a—C10—H10b	111.0115
C17—O2—H2o	106.1 (11)	C10—C11—C12	112.84 (11)
C1—N1—C2	113.14 (10)	C10—C11—H11a	109.4701
C1—N1—C9	105.19 (10)	C10—C11—H11b	109.4716
C2—N1—C9	116.16 (9)	C12—C11—H11a	109.4715
C1—N2—C14	105.33 (9)	C12—C11—H11b	109.4714
C1—N2—C15	113.32 (10)	H11a—C11—H11b	105.8786
C14—N2—C15	116.36 (10)	C11—C12—C13	112.18 (12)
N1—C1—N2	105.37 (10)	C11—C12—H12a	109.4714
N1—C1—H1a	109.4708	C11—C12—H12b	109.4713
N1—C1—H1b	109.4714	C13—C12—H12a	109.4711
N2—C1—H1a	109.4714	C13—C12—H12b	109.4709
N2—C1—H1b	109.4717	H12a—C12—H12b	106.6143
H1a—C1—H1b	113.2791	C12—C13—C14	108.00 (11)
N1—C2—C3	110.68 (10)	C12—C13—H13a	109.4714
N1—C2—H2a	109.4709	C12—C13—H13b	109.4718
N1—C2—H2b	109.4709	C14—C13—H13a	109.4704
C3—C2—H2a	109.4716	C14—C13—H13b	109.4706
C3—C2—H2b	109.4715	H13a—C13—H13b	110.8996
H2a—C2—H2b	108.2321	N2—C14—C9	100.17 (10)
C2—C3—C4	119.73 (12)	N2—C14—C13	117.53 (10)
C2—C3—C8	121.68 (11)	N2—C14—H14	111.415
C4—C3—C8	118.55 (13)	C9—C14—C13	111.83 (10)
O1—C4—C3	121.18 (13)	C9—C14—H14	117.1568
O1—C4—C5	118.71 (12)	C13—C14—H14	99.6523
C3—C4—C5	120.10 (13)	N2—C15—C16	111.17 (11)
C4—C5—C6	120.11 (13)	N2—C15—H15a	109.4717
C4—C5—H5	119.9469	N2—C15—H15b	109.4711
C6—C5—H5	119.9479	C16—C15—H15a	109.4714
C5—C6—C7	120.49 (15)	C16—C15—H15b	109.4709
C5—C6—H6	119.7554	H15a—C15—H15b	107.7171
C7—C6—H6	119.7553	C15—C16—C17	119.99 (11)
C6—C7—C8	119.18 (14)	C15—C16—C21	121.39 (11)
C6—C7—H7	120.41	C17—C16—C21	118.57 (11)
C8—C7—H7	120.4116	O2—C17—C16	121.12 (10)
C3—C8—C7	121.58 (12)	O2—C17—C18	118.62 (11)
C3—C8—H8	119.211	C16—C17—C18	120.25 (11)
C7—C8—H8	119.2111	C17—C18—C19	119.75 (12)
N1—C9—C10	117.23 (11)	C17—C18—H18	120.1267
N1—C9—C14	100.32 (9)	C19—C18—H18	120.1261
N1—C9—H9	111.4609	C18—C19—C20	120.74 (12)
C10—C9—C14	111.72 (11)	C18—C19—H19	119.6301
C10—C9—H9	99.994	C20—C19—H19	119.6303
C14—C9—H9	116.9901	C19—C20—C21	119.43 (12)
C9—C10—C11	107.89 (11)	C19—C20—H20	120.2825
C9—C10—H10a	109.4713	C21—C20—H20	120.2833
C9—C10—H10b	109.4707	C16—C21—C20	121.24 (13)
C11—C10—H10a	109.4714	C16—C21—H21	119.3796
C11—C10—H10b	109.4712	C20—C21—H21	119.3801
			
?—?—?—?	?		

### Hydrogen-bond geometry (Å, °)

**Table:**

Cg1 and Cg2 are the centroids of the C3–C8 and C16–C21 rings, respectively.

**Table:**

*D*—H···*A*	*D*—H	H···*A*	*D*···*A*	*D*—H···*A*
O1—H1O···N1	0.847 (18)	1.967 (17)	2.7096 (14)	145.8 (16)
O2—H2O···N2	0.860 (18)	1.913 (17)	2.6894 (14)	149.5 (17)
C10—H10a···O1^i^	0.96	2.64	3.5666 (17)	163
C13—H13a···O2^ii^	0.96	2.63	3.5458 (17)	160
C10—H10b···Cg1^iii^	0.96	2.94	3.5885 (13)	126
C12—H12b···Cg2^iv^	0.96	2.93	3.7022 (16)	139

Symmetry codes: (i) −*x*+1, −*y*, −*z*+1; (ii) −*x*+2, −*y*, −*z*; (iii) −*x*+2, −*y*, −*z*+1; (iv) −*x*+1, −*y*+1, −*z*.
